# Laboratory Characterization of Asphalt Binders Modified with Styrene Butadiene Rubber (SBR)

**DOI:** 10.3390/ma14247666

**Published:** 2021-12-12

**Authors:** Navid Hemmati, Jihyeon Yun, Mithil Mazumder, Moon-Sup Lee, Soon-Jae Lee

**Affiliations:** 1 Department of Engineering Technology, Texas State University, San Marcos, TX 78666, USA; yiy1@txstate.edu (J.Y.); m_m624@txstate.edu (M.M.); SL31@txstate.edu (S.-J.L.); 2 Korea Institute of Civil Engineering and Building Technology, Goyang 10223, Korea

**Keywords:** SBR, PMA, rutting, viscosity, fatigue

## Abstract

The study describes the laboratory assessment (physical and rheological properties) of the binders (PG 64-22 and PG 76-22) modified with Styrene Butadiene Rubber (SBR), and a comprehensive comparison between these two modified binder types. PG 64-22 and PG 76-22 were used as base binders. Both of the base binders were blended with SBR at four different percentages of content (0%, 4%, 6%, and 8% by the weight of the binder). The base and modified binders were artificially short-term and long-term aged using a rolling thin film oven (RTFO) and pressure aging vessel (PAV) procedures. Superpave binder tests were conducted on the SBR modified binder using rotational viscometer (RV), dynamic shear rheometer (DSR), and bending beam rheometer (BBR). In depth rutting performance was investigated using Multiple Stress Creep Recovery (MSCR). The results of this study indicated that (1) the addition of SBR into both binders increased the viscosity and polymer modified asphalt (PMA) binders observed to have more significant effect on its viscosity property; (2) the higher the SBR content, the better the rutting resistance of the binder and it is observed that the effect is prominent on the control binder; (3) MSCR test results showed that the SBR modified binders improved the binder percentage recovery and found to have a more significant effect on the PG 76-22 binder compared to PG 64-22; and (4) both the control PG 64-22 and PMA PG 76-22 binders resulted in similar trends on the cracking properties and were found to have insignificant effects due to the addition of an SBR modifier.

## 1. Introduction

Due to increased traffic and extreme weather conditions, many defects such as low temperature cracking, fatigue cracking, and high temperature rutting occur [1,2]. Meanwhile, porous asphalt surface has become a widely used asphalt pavement in highways [3]. In addition, asphalt concrete is also used in special pavement areas such as bridge decks, which can be susceptible to dynamic behavior. As more and more special asphalt concretes become popular, there is a need to improve the binder performance. For this purpose, highly viscous modified asphalt binders can generally be considered.

High-viscosity asphalt concrete shows excellent behavior in the field, but a high mixing temperature, around 180 °C–190 °C, is required, and an appropriate temperature for initial quality control (150 °C–170 °C) is also required in situ. To meet these requirements, the selection of a modifier is very important, as quality control and economic costs depend on the modifier. Moreover, even if the performance of asphalt concrete in the laboratory is satisfied, its application in the construction site can be a problem, and the importance of an appropriate modifier selection is increasing. In summary, due to the nature of storage stability, pumping, handling, and workability, various modifiers must be reviewed in many aspects.

There are plenty of modifiers widely used in asphalt binders around the world. In particular, Styrene Butadiene Styrene (SBS), Styrene Isoprene Styrene (SIS) and Styrene Butadiene Rubber (SBR), Crumb Rubber Modifier (CRM), and polyethylene (PE) are commonly used for the asphalt binder modification [4,5]. These materials described are widely utilized and considered to be materials that improve the performance of asphalt binders. They are not only used alone, but can be also used by adding other modifiers and additives to compensate for their benefits and drawbacks. To give an example, incorporation of SIS and ground tire rubber (GTR) into asphalt binders was investigated to determine the high and low temperature rheological properties which illustrated that the blending of GTR and SIS improves the rutting and cracking performance significantly [6]. This means that to improve the performance of the binder, a method of using several modifiers at the same time, supplementing the benefits and drawbacks of the modifiers, can also be considered. SBR is one of the cheap synthetic general-purpose elastomers that is sometimes used as a substitute for Natural Rubber (NR). It is a general-purpose rubber containing about 75% styrene and 25% butadiene joined in a co-polymer. The addition of styrene makes SBR cheaper and improves the bonding and blending capabilities, abrasion resistance, and strength. By the application of SBR as a modifier, viscosity is expected to increase significantly in addition to an improvement in elasticity, as well as adhesive and cohesive properties of the pavement. Due to this excellent cohesion of SBR polymers, the ductility of the asphalt pavement will be increased, which allows more flexibility of the asphalt pavement [7]. In particular, when the SBR content of 3–5% was applied, the performance was improved overall and, in one case, showed an additional improvement, storage stability by adding other materials, meaning that there will be a potential effect by combining other modifiers [8,9,10]. There are limited studies that focus on the viscous effect of SBR content on PG 64-22 and PG 76-22 as a function of different temperature range and other parameters. This means that SBR modifiers should be researched with other binder types, and investigations must be documented in detail.

**Figure 1:**
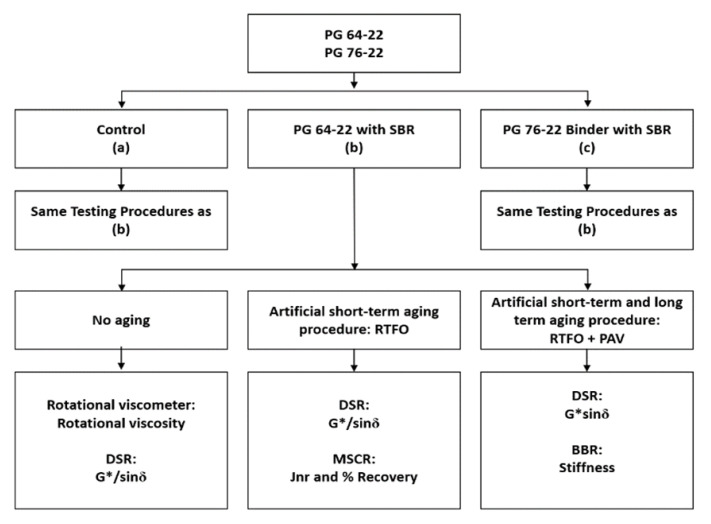
Experimental design procedures used in this study.

The main purpose of this study was to examine the applicability of SBR as a modifier in high viscosity asphalt binder. In particular, the rheological properties of asphalt binder using Styrene Butadiene Rubber (SBR) have been investigated by treating artificially aged using a rolling thin film oven (RTFO) and pressure aging vessel (PAV). The high temperature viscosity properties of the binders were evaluated in the original state through a rotational viscometer (RV) test using four different testing temperatures (at 15 °C intervals from 135 °C to 180 °C). The rutting performance in the original state and after RTFO aging, as well as the fatigue cracking performance at intermediate temperature after RTFO + PAV processes, were evaluated by DSR test. Low temperature cracking performance after RTFO + PAV procedures was evaluated by a BBR test. A flow chart of the experimental design used in this research was displayed in Figure 1. Through this study, a solid understanding of SBR as a modifier for high viscosity asphalt binders can be developed.

## 2. Experimental Design

### 2.1. Materials

**Figure 2:**
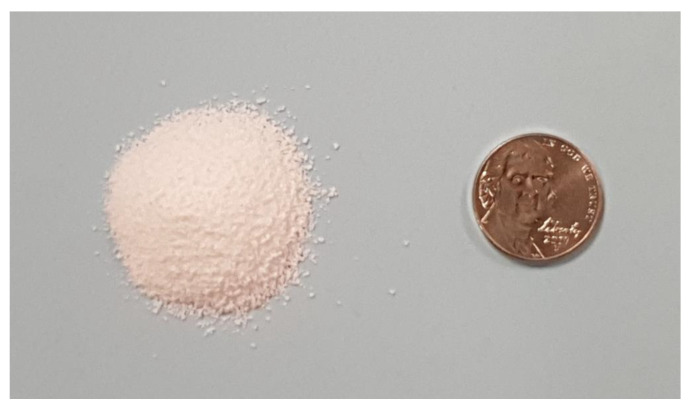
SBR particles.

In this study, performance grade (PG) 64-22 and 76-22 asphalt binders were used as the base binders. Both are most commonly used to produce asphalt mixtures. Their properties are shown in Table 1. Styrene Butadiene Rubber (SBR) can improve the high viscoelasticity required for special asphalt mixtures such as porous mixtures. In particular, the SBR modifier is composed of 95% Styrene-Butadiene Copolymer and 5% of Silicon dioxide. Figure 2 shows the SBR modifier. From an industry perspective, SBR is suitable in terms of stability, reactivity, and toxicological information, except when it is burned. The basic information of SBR is presented in Table 2.

### 2.2. Production and Sampling of SBR Asphalt Binders

The binder was blended using a wet process in which SBR was added directly to the base asphalt binder at the beginning of the modifying process. The SBR modified binder was produced between 170 °C and 190 °C for 60 min at a mixing speed of 4000 rpm in the laboratory [4,11]. Both binders were modified with four different percentages of SBR content (0%, 4%, 6%, and 8% by the weight of the binder). To maintain the consistency of the modified binder during mixing, only one batch of SBR was used in this study.

The control and SBR modified asphalt binders were artificially aged. The first procedure used an RTFO process for 85 min at 163 °C to obtain the short-term aging, followed by a PAV for 20 h at 100 °C to complete the long-term aging. After these aging processes, the tests mentioned in the flow chart were carried out to investigate the properties of SBR modified binders.

### 2.3. Binder Tests for Basic Characteristics

To evaluate workability, which is an important key factor in the special asphalt mixture, the rotational viscosity test was performed at 15 °C intervals from 135 °C to 180 °C, by applying constant velocity of 20 rpm. The 10.5 g binder samples were tested with 27 cylindrical spindles. The time to obtain data was measured at 20 min for each sample.

To measure the viscoelasticity of asphalt binders, the G*/sin *δ* was obtained from the complex shear modulus (G*) and the sine of the phase angle (*δ*). Additionally, by employing these factors of G* and *δ,* fatigue cracking resistance (G*sin *δ*) of asphalt binder was evaluated at a relatively lower temperature of 25 °C. MSCR test is conducted using the DSR for SBR contained binders according to AASHTO T 350-14 specification at 76 °C.

**Figure 3:**
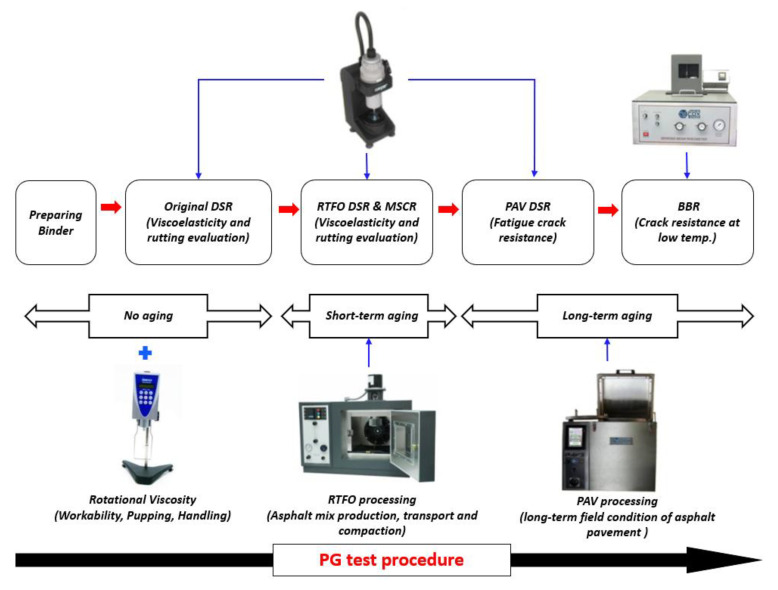
Test procedure in this study.

The stiffness of SBR asphalt binders was evaluated at −12 °C using the Bending Beam Rheometer (BBR) equipment according to the AASHTO T 313. Before conducting this test, the specimen was shaped into the beam (125 × 6.35 × 12.7 mm) and placed on the bath to measure the creep stiffness at the loading time of 60 s, with applying a constant load of 100 g of mass. The overall structure of testing procedure is shown in Figure 3, including the aging process.

### 2.4. Statistical Analysis Method

A statistical analysis was conducted using the Statistical Analysis System (SAS) program to carry out an analysis of variance (ANOVA) and Fisher’s Least Significant Difference (LSD) comparison with an α = 0.05. The major variables contained the SBR contents (0%, 4%, 6%, and 8%) and the asphalt binder types (PG 64-22 and PG 76-22).

The ANOVA was conducted first to decide if significant differences among sample means existed. In the analyses of this study, the significance level was 95 (α = 0.05), suggesting that each finding had a 95% chance of being true. Upon determining that there were differences among sample means using the ANOVA, the LSD was then calculated. The LSD is defined as the observed differences between two sample means necessary to declare the corresponding population means difference. Once the LSD was calculated, all pairs of sample means were compared. If the difference between two sample means was greater than or equal to the LSD, the population means were declared to be statistically different.

## 3. Results and Discussion

### 3.1. Rotational Viscosity

**Figure 4:**
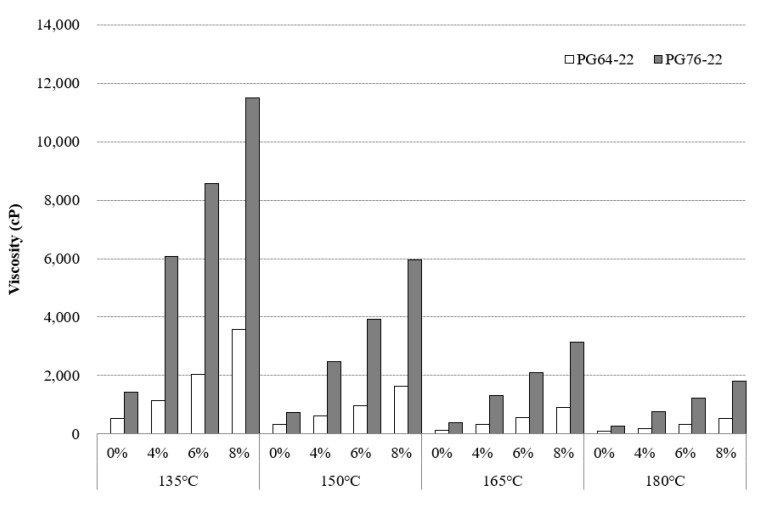
Viscosity of the asphalt binders with SBR at a temperature range of 135 °C to 180 °C.

Specifying the mixing and compaction temperatures of the asphalt binders is crucial because it affects the binder’s capability to be pumped through an asphalt plant, thoroughly coat aggregate in asphalt mixtures, and be placed and compacted to form a new asphalt pavement surface [12]. Figure 4 depicts the standard RV test results of PG64-22 and PG76-22 (original and modified) at 135 °C, 150 °C, 165 °C, and 180 °C.

Figure 4 shows that the addition of SBR significantly increased the binder viscosity regardless of binder types and testing temperatures. At 135 °C, the viscosity of PG64-22 containing 0%, 4%, 6%, and 8% SBR were found to be 538 cp, 1130 cp, 2045 cp, and 3590 cp, respectively. With the same SBR concentrations of PG76-22, the viscosity results were observed 1425 cp, 6060 cp, 8575 cp, and 11500 cp, respectively. With the increments of SBR content at 4%, 6%, and 8%, the viscosity of PG64-22 modified binders increased by 110%, 280%, and 560%, respectively, whereas PG76-22 modified binders were found to have sharp increases of 425%, 600%, and 800% respectively. At 150 °C and 165 °C, a similar trend of increment of viscosity was observed due to the addition of SBR content. At 180 °C, percentage increases of 125%, 280%, and 535% were observed for PG 64-22 at 4%, 6%, and 8% content, respectively. On the other hand, at a similar temperature and SBR content (4%, 6%, and 8%), the percentage increase in viscosity values of PG76-22 modified binders were observed 65%, 78%, and 85%, respectively.

These results show the addition of SBR increases the viscosity of the binders. Based on previous research [13], the characteristics of SBR, such as tackiness, result in an increase in viscosity of the modified binders significantly. High viscosity of the binders was observed to have a negative effect on the workability of the binders during preparation. The high viscosity of the SBR modified binders showed that similar conditions may occur for the mixing process and compaction operation at the jobsite, which can affect the aggregate coating consequently. Even though Figure 4 showed the variation of viscosity values at four different temperatures, according to the Superpave specifications [12], the viscosity of the unaged binder must be less than 3000 cP at 135 °C for good pumping and mixing. By this recommendation, it can be observed that for PG 76-22, the viscosity values of SBR binders at 135 °C does not meet the current maximum requirements by Superpave (i.e., 3000 cP); hence, the percentage content of SBR below 4% can be considered as a good pumping and mixing condition, whereas for PG 64-22, the content of SBR can be increased to 8%.

The statistical significance of the change in the viscosity as a function of SBR content, temperature changes, and binder types were examined, and the results are shown in Table 3. It was observed that the difference among all the SBR binders was found to be statistically significant at 135 °C, 150 °C, 165 °C, and 180 °C in both binders of PG64-22 and PG76-22.

### 3.2. Rutting Property

**Figure 5:**
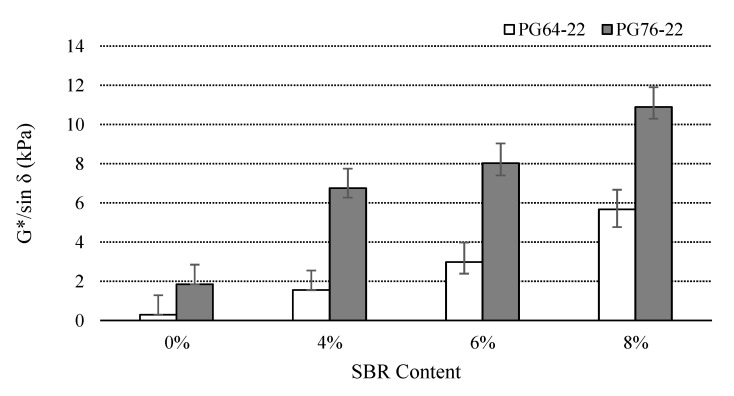
G*/sin *δ* of the original asphalt binders with SBR at 76 °C.

In general, the higher G*/sin δ value from the DSR test means that the binders are more effective against rutting at high pavement temperature [12]. The G*/sin δ values of binders were examined at original and RTFO states at 76 °C. Figure 5 illustrates the results of the conducted tests of the original binders. The addition of an SBR modifier increased the G*/sin δ values for both binder types. Hence, it is evident that the addition of an SBR modifier has a positive effect on the rutting resistance of the binders. The G*/sin δ values of the PG64-22 modified binders increased by 540%, 1030%, and 2000% due to the addition of 4%, 6%, and 8% SBR, respectively. On the other hand, percentage increases of 360%, 420%, and 600% were found for the PG 76-22 binder containing 4%, 6%, and 8%, respectively.

**Figure 6:**
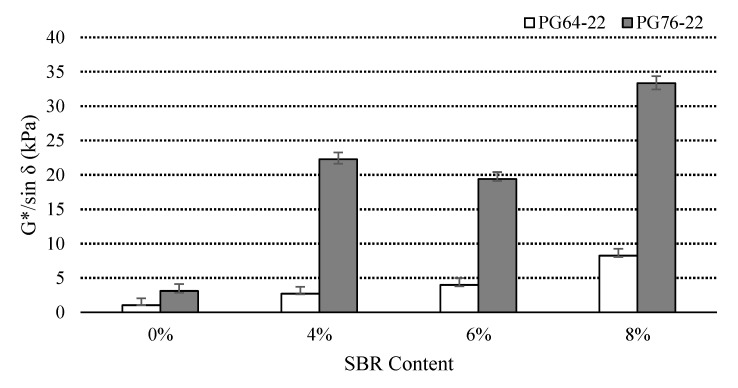
G*/sin *δ* of the RTFO asphalt binders with SBR at 76 °C.

Figure 6 shows the G*/sin *δ* values of all the RTFO binders at 76 °C. The similar trend was observed for the RTFO binders as well compared to unaged binders. The application of SBR into the asphalt binders improved the adhesion and bonding force of binders by increasing viscosity and resulted in more stability and stickiness of the layers in higher temperatures, which caused higher rutting resistance.

According to the Superpave specifications [12], G*/sin δ of all un-aged SBR binders, regardless of PG 64-22 and PG 76-22, exceeded 1.00 kPa at the high service temperature, which prevented rutting. Furthermore, a similar trend was observed for the RTFO of all SBR binders where G*/sin δ of the stiffer RTFO-aged binder exceeded 2.2 kPa at the high service temperature, and prevented rutting. However, based on the specification and data, it is recommended to incorporate more than 4% of SBR with PG 64-22.

Table 4 presents the statistical significance of the change in the G*/sin *δ* at original and RTFO state as a function of SBR content. It indicates that the difference is statistically significant among the binders modified with an SBR modifier.

### 3.3. Multiple Stress Creep Recovery (MSCR) Test

**Figure 7:**
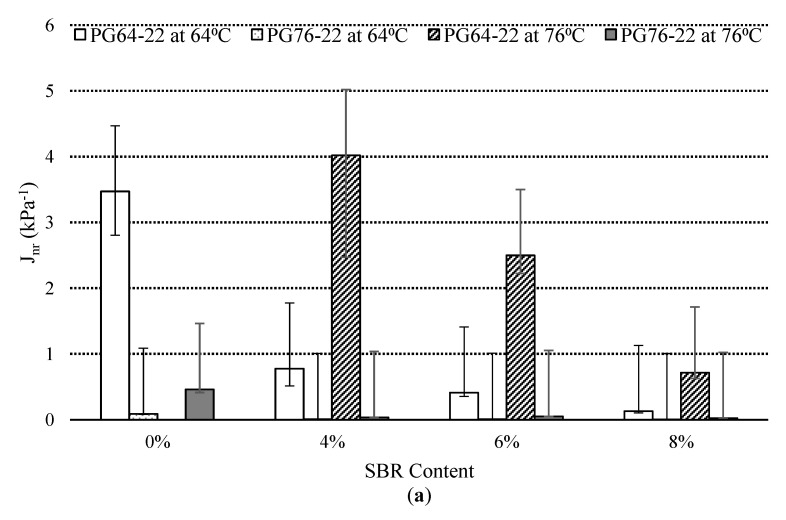

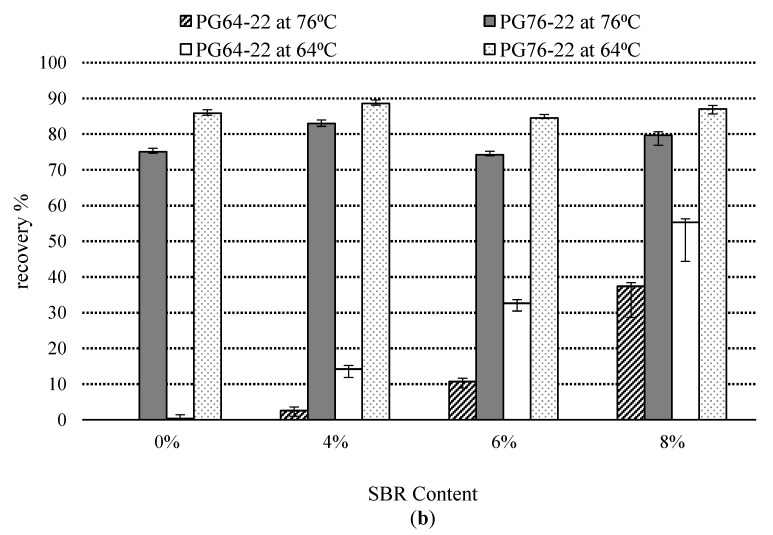
Variations in creep compliance and percent recovery of the SBR modified binder at 64 °C and 76 °C; (**a**) J_nr_ and (**b**) recovery %.

MSCR tests were performed on RTFO aged binders, according to AASHTO TP 70, to evaluate the rutting resistance of the modified binders. Figure 7a,b demonstrates the variation of creep compliance at 3.2 kPa stress level and percent recovery of the SBR modified binders at 64 °C and 76 °C. MSCR test specification specifies a technological advancement in the current PG specification that better describes the high-temperature and stress performance properties of the asphalt binder that can be performed [14]. The non-recoverable creep compliance J_nr_ represents the rutting susceptibility at high temperatures for the modified binders. The %Rec shows the delayed elastic response of the asphalt binder. The variation of creep compliance and recovery percentage at 3.2 kPa stress level of the modified binders at 64 °C and 76 °C are shown in Figure 7a,b for both PG64-22 and PG76-22.

The addition of SBR significantly decreased the J_nr_ values in both binders, as shown in Figure 7a. The addition of SBR into the base binder resulted in a reduction in J_nr_ value of PG 64-22 containing 4%, 6%, and 8% SBR content, by 15%, 8%, and 2.5%, respectively, compared to the control binder at 64 °C. Through investigation of the results of the same binder at 76 °C, reductions of 22%, 12%, and 3% were observed for the binders containing 4%, 6%, and 8% SBR content, respectively. Following a similar trend, PG 76-22 binders containing 4%, 6%, and 8% SBR content, showed a reduction in J_nr_ value of 1.5%, 2% and 1.5%, respectively, at 64 °C. Figure 7b shows the addition of SBR content is effective in increasing the %rec values of modified asphalt binders compared to the base binder. Modified binders containing 8% SBR, showed the lowest J_nr_ and the highest recovery percentage in both binders of PG 64-22 and PG 76-22. For PG 64-22, at 64 °C, the J_nr_ value was found to be 0.4, corresponding to 53% recovery percentage, and at 76 °C the J_nr_ value was observed to be 0.77, corresponding to 33% recovery percentage. The J_nr_ value of PG 76-22 at 64 °C was recorded 0.011 with 86% recovery percentage, whereas at 76 °C, the J_nr_ value was observed to be 0.052, with 77% recovery percentage. The results indicate that binders containing 8% SBR have the greatest recovery rate, after 1 s of creep load compared to the asphalt binders containing 4% and 6%, due to the traffic load. The accumulated strain in the asphalt binder is mainly responsible for the rutting of asphalt pavements. It is worth noting that the percentage recovery due to the addition of SBR modifier was observed to have a more significant effect on the PG 76-22 binder compared to PG 64-22. It is essential in evaluating the recovery of the asphalt binder to investigate its resistance against rutting. Rutting is one of the major stresses or performance parameters in asphalt pavements. It is initiated by the densification and movement of materials under repeated loads and lateral plastic flow under the wheel track. Comparisons of MSCR test results on PG64-22 and PG 76-22, indicates that SBR improved the binder’s viscoelastic characteristic, as an efficient modifier.

Table 5 and Table 6 present the statistically significant results of the MSCR (percentage recovery) and J_nr_, respectively, as a function of the addition of SBR content for a short-term aged binder. For PG64-22 binders, the difference among the SBR binders in terms of percentage recovery was found to be significant, whereas, in general PG76-22, the difference is insignificant.

### 3.4. Fatigue Cracking

**Figure 8:**
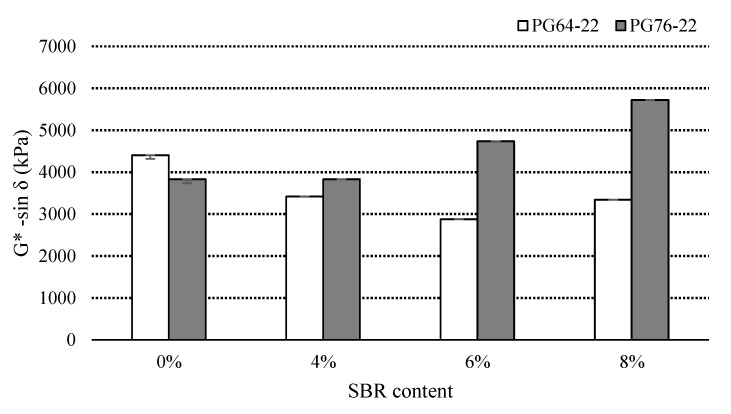
G*sin *δ* of the PAV asphalt binders with SBR at 25 °C.

The G*sin *δ* values from the DSR test indicate the fatigue cracking characteristics, where G* presents stiffness and *δ* is a viscous or elastic indicator. A DSR test was conducted at 25 °C to determine the G*sin *δ* values of the binders (RTFO + PAV residual) to characterize the fatigue resistance. Figure 8 illustrates the G*sin *δ* values of all the binders. The binders of PG64-22 containing 4%, 6%, and 8% SBR were found to be 3420 kPa, 2870 kPa, and 3340 kPa, respectively. PG76-22 binders with similar SBR concentrations were observed to be 3830 kPa, 4740 kPa, and 5720 kPa, respectively.

Table 7 illustrates the statistical significance of the changes in G*sin *δ* due to the SBR content. For PG64-22, the statistical difference among the binders was found to be insignificant. For PG76-22 binders, a statistically significant difference exists between 0% and 4%, as well as between 4% and 8%.

### 3.5. Low Temperature Cracking Property

**Figure 9:**
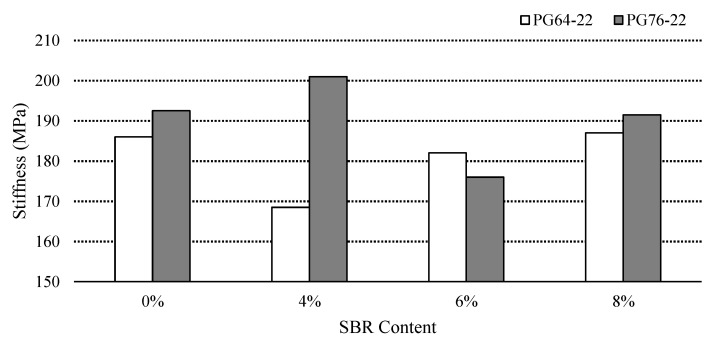
Stiffness of the asphalt binder with SBR at −12 °C.

**Figure 10:**
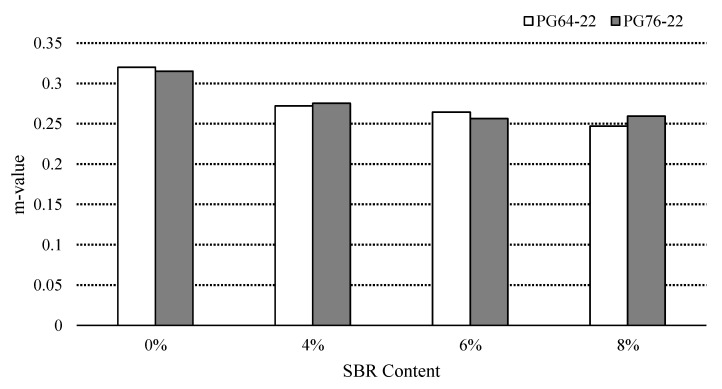
*m*-value of the asphalt binder with SBR at −12 °C.

Superpave asphalt binder specification determines the creep stiffness values up to 300 MPa, and the reduction in stiffness causes a reduction in tensile stress of the asphalt binder; hence, there is less possibility of thermal cracking. BBR tests were conducted at −12 °C to characterize the low temperature cracking properties of the binders. Figure 9 and Figure 10 show the stiffness and *m*-values of SBR modified binders for both PG64-22 and PG76-22. The stiffness of PG 64-22 binders containing 0%, 4%, 6%, and 8% SBR were found to be 187 MPa, 168 MPa, 182 MPa, and 187 MPa, respectively. The stiffness values of PG 76-22 were observed to be 195 MPa, 200 MPa, 176 MPa, and 191 MPa for the binders containing 0%, 4%, 6% and 8% SBR, respectively.

The statistical significance of the stiffness changes and *m*-values as a function of SBR contents were analyzed and are illustrated in Table 8 and Table 9. In general, Table 8 shows the statistical difference of stiffness changes as a function of SBR content changes at −12 °C, which was found to be insignificant.

Table 9 shows the statistical changes of m-vale due to the SBR content. For PG 64-22, the addition of SBR content was found to be statistically significant, except in the difference between 4% and 6% SBR content. For PG 76-22, significant changes in the *m*-values were observed, except changes between the binders containing 4% and 6%, compared to the binder with 8% SBR content.

## 4. Summary and Conclusions

To evaluate the effect of SBR modifier, binders were produced using SBR contents of 0%, 4%, 6%, and 8% by the weight of the base binder. The viscosity property was determined by RV. Rutting, fatigue resistance, and MSCR were evaluated using the DSR equipment at standard temperatures. Moreover, the BBR test was conducted to determine creep stiffness of the control binders and SBR modified binders. Based on the results of these tests, the conclusions are taken for the materials used in this research as follows:

- The viscosity of all the modified binders was increased due to the addition of SBR content. Therefore, it is believed that the workability of the SBR modified binders must be considered during mixing and construction.
- The results of G*/sin *δ* showed a significant improvement in the rutting resistance of the asphalt binders due to the addition of SBR content. The high viscosity and tackiness of SBR modified contributed to maintaining the improved rutting resistance at high temperatures.
- According to the MSCR test results, the addition of an SBR modifier improves the rutting resistance significantly by increasing the percentage recovery of the modified binders.
- From the BBR tests at −12 °C, both PG64-22 and PG76-22 modified binders showed that the addition of SBR content of 4%, 6%, and 8% has no significant effect on stiffness values of the modified binders.
- Based on G*sin *δ* results, the addition of SBR content did not show any significant changes on fatigue resistance.
- Based on G*/sin *δ and* G*sin *δ* results, it is recommended that the incorporation of SBR is considered to obtain high rutting resistance as it does not have any significant effect on the cracking resistance of the binder.
- The conclusions of this study are very limited and cannot be generalized. Further research is needed using different types of asphalt binders and mixtures to draw more general findings.

## Figures and Tables

**Table 1:** Properties of asphalt binders used in this research.

Aging States	Test Properties	PG 64-22	PG 76-22
No aging binder	Viscosity @ 135 °C (cP)	538	1425
	G*/sin *δ* @ 76 °C (kPa)	0.29	1.85
RTFO aged residual	G*/sin *δ* @ 76 °C (kPa)	1.06	3.58
RTFO + PAV aged residual	G*sin *δ* @ 25 °C (kPa)	4400	3830
	Stiffness @ −12 °C (MPa)	187	195
	*m*-value @ −12 °C	0.32	0.315

**Table 2:** The basic physical and chemical properties of SBR used in this study.

Items	Properties
Appearance	Powder
Color	White
Odor	Aromatic
Solubility	Soluble in bitumen and different varieties of oil
Relative density	0.93~0.97
Flash point	246 °C
Auto Ignition Temperature	>388 °C

**Table 3:** Statistical analysis results of the RV test as a function of SBR content (α = 0.05).

Viscosity		135 °C				150 °C				165 °C				180 °C			
		0%	4%	6%	8%	0%	4%	6%	8%	0%	4%	6%	8%	0%	4%	6%	8%
PG64-22	0%	-	S	S	S	S	S	S	S	S	S	S	S	S	S	S	S
	4%		-	S	S	S	S	S	S	S	S	S	S	S	S	S	S
	6%			-	S	S	S	S	S	S	S	S	S	S	S	S	S
	8%				-	S	S	S	S	S	S	S	S	S	S	S	S
PG76-22	0%	-	S	S	S	S	S	S	S	S	S	S	S	S	S	S	S
	4%		-	S	S	S	S	S	S	S	S	S	S	S	S	S	S
	6%			-	S	S	S	S	S	S	S	S	S	S	S	S	S
	8%				-	S	S	S	S	S	S	S	S	S	S	S	S

S: significant. N: Non-significant.

**Table 4:** Statistical analysis results of the G*/sin *δ* as a function of addition of SBR content (α = 0.05).

G*/sin *δ* (Original)		0%	4%	6%	8%
PG64-22	0%	-	S	S	S
	4%		-	S	S
	6%			-	S
	8%				-
G*/sin δ (RTFO)		0%	4%	6%	8%
PG64-22	0%	-	S	S	S
	4%		-	S	S
	6%			-	S
	8%				-

S: Significant. N: Non-Significant.

**Table 5:** Statistical analysis results of the Recovery % as a function of the addition of SBR content (α = 0.05).

Recovery		0%	4%	6%	8%
PG 64-22	0%	-	S	S	S
	4%		-	S	S
	6%			-	S
	8%				-
PG 76-22	0%	-	S	N	N
	4%		-	S	N
	6%			-	N
	8%				-

S: Significant. N: Non-Significant.

**Table 6:** Statistical analysis results of the J_nr_ as a function of the addition of SBR content (α = 0.05).

J_nr_		0%	4%	6%	8%
PG 64-22	0%	-	S	N	S
	4%		-	S	S
	6%			-	S
	8%				-
PG 76-22	0%	-	N	N	N
	4%		-	N	N
	6%			-	N
	8%				-

S: Significant. N: Non-Significant.

**Table 7:** Statistical analysis results of the G*sin *δ* as a function of the addition of SBR content (α = 0.05).

G*sin *δ*		SBR Content			
		0%	4%	6%	8%
PG 64_22	0%	-	N	N	N
	4%		-	N	N
	6%			-	N
	8%				-
PG 76_22	0%	-	N	N	S
	4%		-	N	S
	6%			-	N
	8%				-

**Table 8:** Statistical analysis results of the stiffness as a function of the addition of SBR content (α = 0.05).

Stiffness		0%	4%	6%	8%
PG64-22	0%	-	N	N	N
	4%		-	N	N
	6%			-	N
	8%				-
PG76-22	0%	-	N	N	N
	4%		-	S	N
	6%			-	N
	8%				-

S: Significant. N: Non-Significant.

**Table 9:** Statistical analysis results of the *m*-value as a function of the addition of SBR content (α = 0.05).

*m*-value		0%	4%	6%	8%
PG64-22	0%	-	S	S	S
	4%		-	N	S
	6%			-	S
	8%				-
PG76-22	0%	-	S	S	S
	4%		-	S	N
	6%			-	N
	8%				-

S: Significant. N: Non-Significant.

## Data Availability

The data used to support the findings of this study are included within the article.
